# Laparoscopic Repair of Combined Right Diaphragm and Liver Injuries with a Sharp Object: A Case Report

**DOI:** 10.1155/2013/209494

**Published:** 2013-12-09

**Authors:** M. Kasım Arık, Sükrü Tas, Faruk Özkul, Hasan Şahin, Ozan Karatağ, Muammer Karaayvaz

**Affiliations:** ^1^Department of General Surgery, Faculty of Medicine, Çanakkale Onsekiz Mart University, Floor 2, 17100 Çanakkale, Turkey; ^2^Deparment of Anesthesiology and Reanimation, Faculty of Medicine, University of Çanakkale Onsekiz Mart, Floor 1, 17100 Çanakkale, Turkey; ^3^Deparment of Radıology, Faculty of Medicine, University of Çanakkale Onsekiz Mart, Floor 1, 17100 Çanakkale, Turkey

## Abstract

*Introduction*. Diaphragm injuries develop following penetrating or blunt traumas. The purpose of the case report is to present a 28 year old male patient with stable hemodynamic findings treated with laparoscopic approach following a liver injury combined with a right diaphragm injury caused by a sharp penetrating object. *Case*. 4 cm long transverse laceration was observed near the middle axillary line in the 6th right intercostal space in the examination performed on a 28 year old male patient who applied to the emergency service due to sharp penetrating object injury. Respiratory sounds were decreased in the right side and the examination revealed sensitivity in the abdomen. Elevation in the right diaphragm and hemopneumothorax was monitored in chest X-ray and computerized tomography. Closed subaqueous thorax drain was placed and the patient was taken to the surgery with a right diaphragm injury prediagnosis. Laparoscopic exploration was performed to the patient with stable hemodynamic findings by entering through 10 mm port above the abdomen. 6 cm long injury at the right side of diaphragm and approximately 2 cm deep at the deepest point and 5 cm long linear laceration was observed in the 7th segment of the liver. The diaphragm was repaired laparoscopically with sutures that do not melt on their own. Tampon was applied to the laceration in the liver and bleeding control was performed with suture. Patient was discharged on the 3rd day because he had no problems during postoperative follow-ups. *Result*. No noticed right side diaphragm rupture and possible concomitant visceral organ injuries following a penetrant injury that can cause significant mortality and morbidity should be definitely kept in mind. The detection of right side diaphragm and liver injury is vital with high mortality in case of delayed diagnosis, and direct radiography and computerized tomography are helpful in the diagnosis. Surgical treatment with laparoscopic approach is a method that leads to less hospitalization duration and less pain in cases that are hemodynamically stable.

## 1. Introduction

Diaphragm separating abdomen from thorax can be affected by both thorax and abdominal traumas due to its anatomic localization. Although diaphragm injuries result frequently from blunt and penetrant traumas, spontaneous diaphragm traumas are also reported in literature [1, 2].

Because penetrant traumas can cause smaller injuries, diagnosis of diaphragm injuries becomes difficult and occasionally is not noticed. Diaphragm injuries that develop due to blunt traumas can cause large injuries based on the severity of the trauma. Protective effect of the liver in right diaphragm injuries and herniation of abdominal organs such as stomach and small intestine into thorax in left diaphragm injuries makes the diagnosis of left diaphragm injury easier than right diaphragm injury [3].

No treated diaphragm injuries lead to herniation of gastrointestinal organs into thorax and this situation can cause complications such as strangulation or perforation of translocated organs which may result with high mortalities [4, 5]. Therefore surgical repair should be performed without any delay following the diagnosis of diaphragm injuries. Repair is performed with thoracic or abdominal approach and with open or laparoscopic techniques.

The aim of our study is to present right side diaphragm injury developed as a result of penetrant trauma and repaired with laparoscopic method, together with literature.

## 2. Case Report

**Figure 1:**
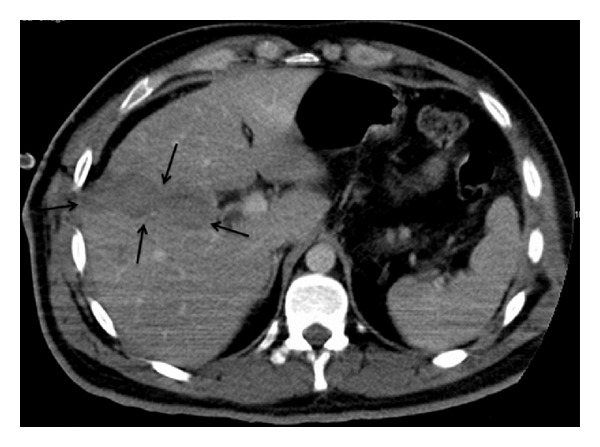
CT imagination of liver rupture.

**Figure 2:**
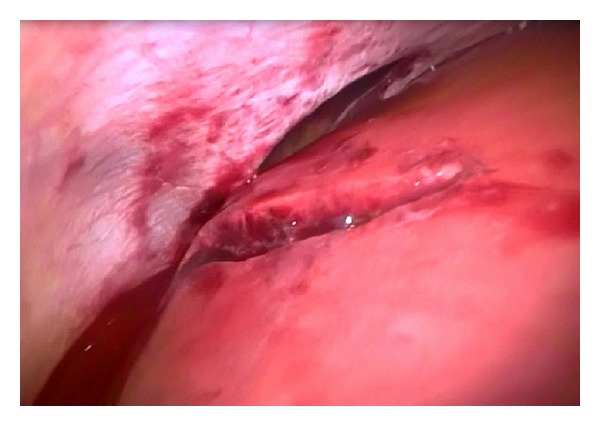
Laparoscopic view of diaphragm rupture and liver injury.

28 year old male patient applied to the emergency service with a complaint of sharp-cutting object injury. Physical examination of the patient revealed approximately 4 cm long transvers laceration at the point where the 6th right intercostal space and midaxillary line intersect. A decrease in the right thorax respiratory sounds was detected when listened. A wide sensitivity was observed during abdominal examination. Elevation in right diaphragm and pneumothorax in the right lung was observed in the posterior-anterior lung radiography of the patient. Free liquid in right upper quadrant of the abdomen was detected in USG and liver injury in CT (Figure 1). After right thorax closed subaqueous drainage was applied immediately, the patient was taken to the surgery with a right diaphragm prediagnosis. In the surgery performed with laparoscopy method, approximately 5 cm long and 2 cm deep injury were detected in the 7th segment of the liver with the aid of 30° cameras (Figure 2). Also a 6 cm injury opening to thorax was monitored in right thorax. Lung and thorax tubes were controlled with the help of camera forwarded from region of injury to thorax. No pathology was detected in the thorax. Injuries in diaphragm and liver were repaired primarily. Respiratory distress was solved after two days. The thorax drainage of the patient with normal clinical follow-ups was pulled on the 3rd day and he was discharged on the 4th day. There were not any complaints on the 15th day and 2nd month follow-ups after surgery and physical examination and radiological findings were normal.

## 3. Discussion

Diaphragm injuries were first defined by Sennertus in 1541 and the first surgical repair was performed by Rolfi in 1886. Although limited numbers of spontaneous diaphragm injuries are reported in literature, diaphragm injuries occur frequently following assault, falling on top of a pointy object from high and other penetrant traumas. Although the percentages reported differ, 75–87% of diaphragm injuries result from penetrant causes. Diaphragm injuries related to penetrant traumas are more frequently encountered in locations with high crime rates and those related to blunt traumas are seen more frequently in regions where agriculture and construction sectors predominate and traffic accidents rates are high.

Although it has been reported that diaphragm injuries on the right side were less frequently encountered, post-mortem studies showed that actually injuries on both sides were seen in equal numbers. This situation is related to the increase in mortality with big vascular structures and liver injuries accompanying the case on the right side. The detection of right side diaphragm injuries is much harder than that of left side injuries because in early period radiological examinations, herniation of visceral organs into thorax in right diaphragm injuries cannot be monitored easily due to the liver acting as a tampon [6].

While tearing in diaphragm in penetrant injuries is related to the size of the object causing the injury, in blunt traumas, it is more related to pressure and energy transfer. Therefore, much larger injuries occur in blunt traumas. Morbidity and mortality in diaphragm injuries change due to primary injuries as well as coexisting organ injuries [7]. In literature, it has been reported that lung injuries accompanied it [8].

Diaphragm injuries are evaluated in 3 clinical stages. In diaphragm injury staging model defined by Carter, 1st stage covers the period from the start of traumatization to the recovery of the injury. While cardiovascular problems and respiratory distress are the main symptoms in this stage, findings related to other organ injuries are added to the picture. 2nd stage is the interim period entered with the start of recovery. In this stage minor symptoms of herniation developed due to not noticed diaphragm injuries predominate. And the 3rd stage, the last step in diaphragm injuries is represented by late situations such as incarceration, strangulation and perforation developed due to visceral organ herniation. Naturally mortality and morbidity are observed frequently in the last phase.

The first diagnosis method for diaphragm injuries is direct radiography. But in right side diaphragm injuries, more advanced methods such as computerized tomography may be needed frequently. It is reported that computerized tomography has 83% sensitivity because it can monitor radiological features related to abdominal organ advancement into the thorax and it can more clearly detect eventration in the diaphragm [9].

In this case report, the patient had the chance of early intervention with the diagnosis established with the CT taken due to continuing respiratory distress despite the inserted thorax tube. The most important stage in diaphragm injuries is stage 1 and it is essential not to skip the injury. In this case with early diagnosis, we were able to detect it in the 1st stage.

In cases where the diagnosis is not clear despite advanced radiological examinations, patients may be diagnosed during the 1st stage in rates up to 95% by performing laparoscopy and thoracoscopy and when necessary surgery (VATS). But it should be kept in mind that although diaphragm injury may be detected, intraabdominal additional organ injuries may not be noticed when only VATS is performed. Nowadays, advances in laparoscopic surgery and anesthesia increase diagnosis rate and enable the treatment in the same stage.

In our case we preferred to perform diaphragm repair with laparoscopic method when we considered the hemodynamic stability. In cases where laparoscopic method is used, mostly a clearer view is achieved with less ecartation due to insufflation. In open surgery, angle camera assistance can make the introduction to locations hard to reach, much easier. Situations like infections, long hospital stay, pain, and rate of incisional hernia development in late period seen in patients to whom open surgery with laparoscopic repair is performed are minimalized. Length of hospital stay in open surgery is 5–16 days when compared with 2–7 days in laparoscopic surgery [10, 11]. The patient of our case was discharged on the 4th day. The use of laparoscopy in penetrant injuries is increasingly becoming popular and it is reported that its efficiency in a meta-analytic study is between 67% and 100% [12]. There are authors claiming the superiority of thoracoscopy for the injuries on the left side and of laparoscopy for the injuries on the right side (223). Another superiority of laparoscopic approach to thoracoscopy is that it is possible to diagnose and treat co-existing intra-abdominal organ injuries. In our case, liver injury was detected also and in the same session, primary repair of liver was performed.

## 4. Result

As a result, it should not be forgotten that no noticed diaphragm injuries can cause respiratory problems, herniation of intra-abdominal organs, and hernia complications with high mortality rates such as strangulation-perforation on the following days. Laparoscopic surgical treatment in patients with appropriate hemodynamics decreases the length of hospital stay in postoperative period and enables a more comfortable postoperative period.
